# Analysis of misdiagnosed cases of hemorrhagic fever with renal syndrome in children: two cases and literature review

**DOI:** 10.1186/s12882-019-1562-0

**Published:** 2019-10-23

**Authors:** Li Zhang, Qing-shan Ma, Yan Zhang, Bai-chao Sun, Leng-yue Zhao

**Affiliations:** Department of Pediatrics Nephrology, First Hospital, Jilin University, Changchun, 130021 Jilin China

**Keywords:** Hemorrhagic fever with renal syndrome, Children, Atypical, Hantavirus

## Abstract

**Background:**

Hemorrhagic fever with renal syndrome (HFRS) is an acute disease caused by hantavirus infection and is clinically characterized by fever, various hemorrhagic manifestations and transient renal and hepatic dysfunctions. Although various cases of HFRS have been reported, cases in children have rarely been described. Herein, we report two atypical cases of HFRS in children without distinctive manifestations and typical disease clinically progresses.

**Case presentation:**

Patient 1 was a 11-year-old girl who attended our clinic for fever accompanying with acute renal failure, proteinuria and decreased level of complement 3 (C_3_) and thrombocytopenia without any hemorrhagic manifestations, acute glomerulonephritis was suspected first, especially lupus nephritis. Patient 2 was misdiagnosed as encephalitis at local hospital because of fever and headache for 4 days. With elevated liver transaminases, proteinuria and normal cerebrospinal fluid examination, HFRS was taken into consideration. Both of the two cases were supported and confirmed by serological test for Hantavirus.

**Conclusions:**

Clinical manifestations of HFRS in children often presented atypically and were milder than adults. Febrile disease accompanying with thrombocytopenia may lead to the suspected diagnosis of HFRS.

## Background

HFRS is a zoonosis caused by viruses that belong to the genus Hantavirus [1], which is prevalent among European and Asian countries [2]. It typically presents itself with the triad of fever, hemorrhage, and acute kidney injury [3]. The disease may manifest itself in five distinct phases that are characterized by the presence of fever, hypotension, oliguria, diuresis and convalescence. While most reported cases have been reported in adults [4, 5], children with this illness may have atypical presentations that lead to delayed or missed diagnosis. We herein reported two children with HFRS, whose diagnoses were both delayed due to atypical presentations.

## Case presentation

### Patient 1

A 11-year-old girl from a rural village attended our clinic with a history of fever and cough since 6 days prior. She also complained of muscle weakness and fatigue, and had a decrease in urine output and swelling eyes. Physical examination revealed with mild eyelids and facial edema, facial blushing, oral ulcer, throat congestion and bilateral vertebral angle tenderness. Family history was significant for father who recovered from acute kidney injury with unknown etiology 1 year ago.

Vital signs were stable (BP 96/80 mmHg, P 106/min, R 24/min) and laboratory investigations were depicted in Table 1. In summary, she got leucocytosis (20.0 × 10^9^/L, with 64% neutrophils), normal hemoglobin and thrombocytopenia (66 × 10^9^/L) shown in routine blood analysis. She got elevated hepatic transaminases (ALT 228 U/L, AST 235 U/L) and lactate dehydrogenase (764 U/L). In addition, her blood urea nitrogen and creatinine were 13.32 mmol/L and 104 μmol/L respectively, which were elevated than normal, and the eGFR declined to 64 ml/min. Her albumin (33.7 g/L) and total calcium (1.93 mmol/L) both declined. Routine urine analysis showed proteinuria (3+) and hematuria (2+) with normal number RBC in HPF. Renal ultrasonography showed swelling of both kidneys, increase in echogenicity and reduced corticomedullary differentiations without urinary lithiasis.

**Table 1:** Overview of laboratory investigations in Patient 1

Laboratory finding	Normal range	Clinic	Day 1	Day 2	Day 4	Day 6	Day 8
WBC (× 10^9^/L)	3.50–9.50	20.00	20.78		10.62	7.17	
GR%	40–75	64	37		58	46	
GR# (× 10^9^/L)	1.80–6.30	12.3	7.70			3.31	
LY%	20–50	21.9	47		25	32	
LY# (×10^9^/L)	1.10–3.20	4.38	9.83			2.31	
HGB (g/L)	115–150	155	146			144	
PLT (×10^9^/L)	125–350	66	106			473	
ALT (U/L)	9.0–50.0	228	141				22
AST (U/L)	15.0–40.0	235	141				28
LDH (U/L)	120–250	764	660		462		293
HBD (U/L)	72.0–182.0	537	517				236
Ca (mmol/L)	2.25–2.67	1.93	/	1.97	/	/	2.42
ALB (g/L)	40.0–55.0	33.7	32.4				38.1
Ferritin (μg/L)	20.0–200.0	/	/	1291.5			149.4
C_3_ (g/L)	0.9–1.5	/	/	0.73	0.99		
CRP (mg/L)	0.00–3.00			14.2	10.60		
BUN (mmol/L)	2.6–7.5	13.32	12.47	11.44		8.72	5.50
Scr (μmol/L)	50–80	104	96.2	99.8	122.7	75.3	57.9
eGFR (ml/min)	80–120	64	69		54.3	88.5	
Proteinuria (g/24 h)	-, 0–0.15	3+		2+	1.24	–	0.02
Hematuria (/HPF)	-, 0–3	2+, RBC 1.3/HPF					
MP - Ab	< 1:40			1:80			
IgM capture ELISA anti- Hantaan virus	–	/	/	/	/	+	

*MP*Mycoplasma pneumoniae

*Ab* Antibody

*ELISA* Enzyme-linked immuno sorbent assay

On the basis of these, it seemed like some kind of acute glomerulonephritis acquired after infection. In order to identify the diagnosis, she was admitted to our department. As she was an adolescent girl with fever, oral ulcer, thrombocytopenia, proteinuria, hematuria, and especially with C_3_ levels declined which was found on the second day after hospitalizing, systemic lupus erythematosus (SLE) was taken into consideration first. Besides, her father had fallen into renal failure before, which provided Alport Syndrome as another speculation. Another abnormal index was elevated ferritin, combined with changes in routine blood analysis, which made bone marrow puncture be needed. Because of the benign prognosis of most post-infection glomerulonephritis, renal biopsy is not necessary in most cases. However, her urine protein quantity was 1.24 g/24 h, which was rather high for children, renal biopsy was under consideration. The titer of Mycoplasma pneumoniae (MP) antibody increased to 1:80. MP infection could also cause extrapulmonary injury, such as hepatic function impairment and nephritis. Along with fever, she got transient nausea and vomiting on the third day. Antibiotics and supporting treatment were given to her. Taking into account the diseases mentioned earlier, we were going to give her kinds of invasive examination. Fortunately, the abnormal indexes recovered gradually after symptomatic treatment, shown in Table 1. On day five, she no longer got fever. On day six serum was found positive for Hantaan virus IgM antibody which using the method of IgM capture ELISA.

When we repeated the history regarding possible rodent exposure, her family stated that there were a lot of rodent activity and patients diagnosed as epidemic hemorrhagic disease in their place of residence. During the disease evolution, febrile stage and oliguria stage appeared at the same time, and diuresis stage appeared on the 5th day without hypotension. She recovered well with supportive treatment without residual complications.

### Patient 2

A 13-year-old girl was transferred from a local hospital to our department with a diagnosis of encephalitis because of fever and headache for 4 days. She also complained of orbital and abdominal pain. Upon presentation, her vital signs were normal (BP 110/70 mmHg, P 100/min, R 24/min) and physical examination included facial blushing when fever, pale face and palpebral conjunctiva, throat congestion, splenomegaly (spleen located in subcostal arch 2 cm) and negative nervous system examination.

Cerebrospinal fluid examination taken in clinic was normal (protein 0.24 g/L, glucose 6.38 mmol/L, chlorine 121.7 mmol/L, Pan’s reaction: negative, WBC 7 × 10^6^/L, RBC 0 × 10^6^/L). Routine blood analysis in clinic showed leucocyte with left shift (5.60 × 10^9^/L, with 89% neutrophils), mild anemia (HGB 97 g/L) and normal quantity of platelet (172 × 10^9^/L) which declined to 104 × 10^9^/L for the next day. Liver transaminases (ALT 143 U/L, AST 96 U/L) and lactate dehydrogenases (638 U/L) were both elevated. Urine showed proteinuria (3+), meanwhile both blood urea nitrogen and creatinine were normal. Renal imaging also showed renal swelling (Left: 125 mm × 59 mm; Right: 124 mm × 57 mm; Normal range: Left: (93.2–105.2) mm × (47.6–54.6) mm; Right: (84.1–94.9) mm × (44.7–51.9) mm) and poor corticomedullary differentiation. In addition, the ferritin was 444.6 μg/L which was elevated and heteromorphic lymphocyte (17%) was seen in blood smear examination. The titer of MP antibody increased to 1:80. Detailed laboratory investigations were depicted in Table 2.

**Table 2:** Overview of laboratory investigations in Patient 2

Laboratory finding	Normal range	Clinic	Day 1	Day 2	Day 3	Day 6	Day 10
WBC (×10^9^/L)	3.50–9.50	5.60	5.69	7.84	10.01	5.66	6.30
GR%	40–75	89	45	24	21	41	38
GR# (×10^9^/L)	1.80–6.30		2.55	1.88	2.14	2.31	2.37
LY%	20–50	8	31	50	66	48	56
LY# (×10^9^/L)	1.10–3.20		1.76	3.89	6.62	2.72	3.53
HGB (g/L)	115–150	97	89	91	81	81	87
PLT (×10^9^/L)	125–350	172	104	146	111	190	409
ALT (U/L)	9.0–50.0			143			
AST (U/L)	15.0–40.0			96			
LDH (U/L)	120–250			638			
HBD (U/L)	72.0–182.0			441			
Ca (mmol/L)	2.25–2.67		1.87	1.95	2.01	1.83	2.17
ALB (g/L)	40.0–55.0			34.2			
Ferritin (μg/L)	20.0–200.0			444.6			
CRP (mg/L)	0.00–3.00		11.90			1.76	
BUN (mmol/L)	2.6–7.5		3.41	3.38	2.73	1.86	3.32
Scr (μmol/L)	50–80		44	58.6	50	48.4	35.6
Proteinuria (g/24 h)	-, 0–0.15		2+	1+	3+		
MP - Ab	< 1:40			1:80			
IgM capture ELISA anti- Hantaan virus	–	/	/	/	+		

*MP* Mycoplasma pneumoniae

*Ab* Antibody

*ELISA* Enzyme-linked immuno sorbent assay

The patient presented headache and fever in autumn, which easily led pediatrician to take encephalitis into consideration first. However, with the normal cerebrospinal fluid examination in clinic and negative nervous system examination in our department, it was ambiguous to make the diagnosis as encephalitis. Due to mild anemia and thrombocytopenia, accompanying with elevated ferritin and heteromorphic lymphocyte, we had to decide whether or not to do a bone marrow puncture. In addition, with damage in hematological system accompanied with proteinuria occuring in an adolescent girl, we should also pay attention to identification of SLE. As her complain of orbital pain, HFRS was also taken into consideration. Despite the possibility, the consultation from infectious disease department did not consider HFRS at first.

HFRS was later supported and confirmed by her serological test for Hantaan virus on the 3rd day, which was positive. Then she was transferred to infectious disease department for treatment including antiviral drugs and supporting treatment. During the disease evolution, diuresis stage appeared on the 7th day without oliguria stage and hypotension. Although she discharged hospital from another department, she was under follow-up by pediatrician. After accepting supportive therapy, she also recovered well.

## Discussion and conclusions

HFRS is an acute illness caused by Hantavirus in the family Bunyaviridae. It is a kind of zoonosis. Infection of humans by different viruses presents different clinical severities. The most commonly found species in Western-Europe is Puumala virus [6], which usually caused a milder form HFRS. Especially, infection among children are much lighter than adults [7]. While in Asia, mainly in China, Hantaan virus (HTNV) and Seoul virus (SEOV) are the common species [8, 9], that often cause the more severe form HFRS. It is notable that a novel European hantavirus, Sochi virus, has been discovered which causes severe courses of hantavirus disease with a high mortality [10]. Besides HFRS, Hantavirus could cause hantavirus cardiopulmonary syndrome (HCPS) or hantavirus pulmonary syndrome (HPS).

Human infection occurs primarily through via virus-containing, aerosolized rodent excretions such as urine, feces, or saliva [11]. It is more prevalent among rural inhabitants where rodent infestations are common. Rodents act as natural habours of the Hantavirus and can transmit the disease through direct innoculation by biting. Victims can also be infected via contact with rodent dropplings that are contaminated with the virus [12]. Although both of our patients denied being bitten by mice, they might have contacted contaminated dropplings without notice. In addition, the father of our first patient had similar clinical presentation a year ago supports the postulation that they both might have been exposed to Hantavirus in the same environment.

The disease constellations of HFRS include fever, various hemorrhagic manifestations and transient hepatic/renal functions impairments [13]. It generally progresses in five distinct phases, including fever, hypotension, oliguria, diuresis and convalescence [14]. The presence of endothelial damages, as evidenced by capillary dilatation and leakage, is considered to be a hallmark of the hantavirus infection [15]. Although the exact pathomechanisms are unclear, the clinical conundrum of marked cytokines production, kallikrein-kinin and complements activations, and elevated levels of circulating immune complexes suggest the pivotal roles of immune responses to the infection.

Although hantavirus infection is a global health issue [16], about 90% of reported cases are from China [17]. Even though childhood infections were not common among reported cases, this phenomenon might be due to under-diagnosis, as the clinical manifestations of HFRS in children can be atypical [12]. Meanwhile, it is reported that the clinical impression of HFRS is lighter in children than in adults [7]. In their review, they concluded three aspects of difference. First, in clinical symptoms aspect, adults usually had arthralgia, muscle pain and visual disturbances, while abdominal pain and vomiting was more common in children than in adults. Second, in aspect of physical signs, transient hypertension was more common in the children. Third, in laboratory tests almost all adults had significant leukocytosis, whereas in children the most common laboratory finding was thrombocytopenia. However, a recent analysis pointed different view [18]. Despite some certain differences among symptoms, acute kidney injury (AKI) and thrombocytopenia occurred at similar frequencies and severity in both children and adults. Another research claimed that the presence of thrombocytopenia is highly sensitive and specific for detecting patients with hantavirus infection [19]. Platelet count was also a predictor and marker of disease severity and progression [20, 21].

In childhood, hantavirus infection is not an common etiology of acute kidney injury. The diagnosis of HFRS is based on clinical manifestation, epidemiological data and laboratory tests. However, in cases with mild or atypical clinical symptoms, it is hard to diagnosis only based on clinical symptoms or signs. Under these situations, laboratory tests turn out to be of great importance. The most common serological tests are indirect IgM and IgG ELISA as well as IgM capture ELISA [22, 23]. Besides, the hantavirus infection can be confirmed by detection of hantavirus genome in blood or serum samples by RT-PCR. Consider the economic, we adopted the method of IgM capture ELISA in our center. Both of two patients were confirmed by positive result for Hantaan virus IgM antibody. Still, it often takes a long time to get serological tests result to support a presumptive diagnosis. When we reviewed the course of diagnosis and treatment, we tired to work out which symptoms led to the suspected diagnosis. In Patient 1, besides of fever, she demonstrated acute hepatic and kidney injuries associated with thrombocytopenia and low C_3_ level, but she did not have any clinical feature of hemorrhage, nor did her physical signs reveal any hypertension. Besides, both fever and thrombocytopenia could happen in either blood disease or autoimmune disease, which made us almost give her a bone marrow puncture and a kidney biopsy. Also she did not presented in typical five different phases. Therefore, her hantavirus infection was not ascertained until the serological results were later confirmed. The delay in serological tests caused a dilemma in initial examination and treatment. Similarly, in Patient 2, her presentation of fever with severe headache had also rendered the diagnosis of HFRS a challenge during her initial clinical course. Although there was nausea and vomiting in Patient 1 and abdominal pain in Patient 2, these symptoms were not of specificity. Besides, hybrid infections often occur in children, such as MP infection in both patients, which could also make us confused. From former literature and our case presentations, fever accompanying with thrombocytopenia in both patients might lead to suspected diagnosis of HFRS. It gave us a reminder to consider HFRS and distinguish from other diseases.

The transient decline of C_3_ level in Patient 1 might support that the immune mechanisms play an important role in the pathogenesis of HFRS. Their platelet counts recovered soon presented that they were under a milder disease course as well. There was splenomegaly, anemia and heteromorphic lymphocyte in Patient 2, that probably due to inflammation in the spleen. The inflammation in the spleen was reported in hantavirus infections in hosts [24]. However, that could not be identified, as we did not do bone marrow puncture nor any further examinations. It was a limitation that we did not do renal biopsy for them, so that we could not recognize the renal pathology of HFRS. Since they had complete recovery, it was benefit for patients not undergoing invasive examinations.

In case of hantavirus infection there is no specific treatment. Generally the primary treatment of hantavirus infection is mainly supportive. Renal function recovers completely within a few days in most cases. Hemodialysis, oxygen therapy and shock therapy are sometimes needed. However, when it came to the conditions with deadly hantavirus infections, there is an increasing demand to develop special therapy. Under the known pathogenesis, drugs that are known to influence increased capillary permeability, such as kinases, angiopoietin 1 and sphingosine 1­phosphate are in clinical trials [25]. Luckily, both of our patients suffered mild manifestation and recovered fully without any residual damage after supportive treatment. What needs to be explained is that our Patient 2 accepted antiviral drug (ribavirin) in infectious disease department. Controversy has arisen in whether or not using ribavirin in treatment of HFRS. A previous trial, in which prospective, double-blind, concurrent, placebo-controlled method was used to observe the effect of intravenous ribavirin therapy of HFRS in China, reported that both morbidity and mortality significantly declined [26]. However, a recent trial for the treatment of HFRS in Russia indicated that intravenous ribavirin did not alter viral load kinetics [27]. Consider the side effect of using ribavirin in children, we do not recommend that children are treated with ribavirin for HFRS. The best defence would be to prevent serious infection from occurring. Since there are no FDA-approved vaccines or treatments for these hantavirus diseases by now [28], it is urgent for the development of vaccines in postexposure prophylactic.

Our patients illustrate how HFRS in children is a clinical entity with multiple manifestations, and high index of suspicions are vital in making the correct diagnosis. When facing unexplaned fever accompanying with thrombocytopenia, pediatricians should take HFRS into consideration. Serological test should be adopted for diagnose in time. Early acknowledge could avoid unnecessary invasive examinations and treatment delay.

## Data Availability

The datasets used analyzed during the current study are available from the corresponding author on reasonable request.

## Abbreviations

AKI: Acute kidney injury
C_3_: Complement 3
eGFR: estimated glomerular filtration rate
ELISA: Enzyme-linked immuno sorbent assay
FDA: Food and Drug Administration
HCPS: Hantavirus cardiopulmonary syndrome
HFRS: Hemorrhagic fever with renal syndrome
HPS: Hantavirus pulmonary syndrome
HTNV: Hantaan virus
MP: Mycoplasma pneumoniae
RT –PCR: reverse transcription -PCR
SEOV: Seoul virus
SLE: Systemic lupus erythematosus
